# School grounds and physical activity: Associations at secondary schools, and over the transition from primary to secondary schools

**DOI:** 10.1016/j.healthplace.2016.02.004

**Published:** 2016-05

**Authors:** Flo Harrison, Esther M.F. van Sluijs, Kirsten Corder, Andy Jones

**Affiliations:** a Norwich Medical School & UKCRC Centre for Diet and Activity Research, University of East Anglia, Norwich, UK; b MRC Epidemiology Unit & UKCRC Centre for Diet and Activity Research, University of Cambridge School of Clinical Medicine, Institute of Metabolic Science, Cambridge Biomedical Campus, Cambridge, UK

**Keywords:** Physical activity, Environment, Secondary school, Primary school, Audit, Adolescents

## Abstract

This paper aims to further understanding of the physical environments of secondary schools and their associations with young peoples' physical activity. Accelerometer-derived physical activity measurements from 299 participants in the SPEEDY study (Norfolk, UK) were obtained from baseline measurements (age 9–10 y) and +4y follow-up. These were linked to objective measures of primary and secondary school environments as measured by the SPEEDY grounds audit tool. We saw considerable differences in the nature of school grounds between primary and secondary schools. Cross-sectional associations were seen between active travel provision scores and commuting time moderate-to-vigorous physical activity (MVPA) for 13–14 year old boys and adolescents living further from school. However, few associations were seen between changes in school grounds scores and changes in school-based MVPA.

## 1 Background

Schools are important settings for the promotion of children's physical activity. Through commuting, break times, and physical education lessons they provide regular opportunities for children to be active (Ridgers et al., 2006). Past work has found that children can acquire up to 40% of their daily moderate-to-vigorous physical activity (MVPA) during school break times (Ridgers et al., 2006), and between 25% and 40% during travel to and from school (van Sluijs et al., 2009). Previous work has highlighted how alterations and additions to the physical school environment can increase children’s activity levels (Harrison and Jones, 2012), and that the supportiveness of primary school physical activity environments is positively related to children's school-time activity levels (Jones et al., 2010).

Children's physical activity is known to decline as they age, and the transition to adolescence (Dumith et al., 2011), coinciding with the move from primary to secondary education, is seen as key point at which to intervene (Cale and Harris, 2006). MVPA has been shown to decline more strongly over these ages at school lunch times, during which school grounds are key locations for physical activity, than at other periods of the school day (Brooke et al., in press). There is some evidence that changes in the environmental supportiveness of schools between primary and secondary settings are associated with changes in physical activity. De Meester et al. (2014) found that young people's weekday step counts increased if the quantity of schoolyard facilities and equipment was higher at secondary schools than primary schools. Despite this, much work on activity promotion through the design of school grounds has focused on primary schools. A recent review of the role of school playgrounds in children's physical activity included 33 papers, of which only two were set in secondary schools (Broekhuizen et al., 2014). These cross-sectional analyses found associations between increases in the number of facilities in the school grounds and increased self-reported physical activity during recess (Haug et al., 2010, Haug et al., 2008). Broekhuizen et al. (2014) concluded that further work is needed to explore if and how secondary school grounds can be adapted to promote physical activity in older children and adolescents.

The assessment of school grounds may be conducted via questionnaire surveys of staff or students, as used by De Meester et al. (2014), or objectively through the use of systematic observational audits. The audit approach requires the development of an audit tool through which standardized measurements of characteristics such as the presence of individual items of equipment, the standards of maintenance of facilities, and the more subjective feel of an area, may be taken across different settings (Brownson et al., 2009). Such an audit tool was developed to assess the suitability of school grounds for physical activity as part of the Sport Physical Activity and Eating Behaviour, Environmental Determinants in Young People (SPEEDY) study (Jones et al., 2010). The validity and reliability of the SPEEDY school audit tool was tested in primary schools, and showed that the supportiveness of primary school grounds was related to children's school-time MVPA (Jones et al., 2010). The SPEEDY audit tool has since been adapted to assess the supportiveness of primary school environments for physical activity around the world (Katzmarzyk et al., 2013).

Given the need to understand how secondary school grounds can support young people's physical activity, this study has three aims; (1) to assess if and how the supportiveness of school environments for physical activity change between primary and secondary schools, (2) to assess the cross-sectional association between the secondary school environment as assessed by the SPEEDY school audit tool and young people's school-based MVPA, and finally (3) to assess the association between change in children's school based MVPA and change in school physical activity environment supportiveness across the transition from primary to secondary school. These aims will be met through analyses of data collected as part of the SPEEDY study in Norfolk, UK.

## 2 Methods

### 2.1 Recruitment and data collection

The SPEEDY study (Sport, Physical activity and Eating behaviour: Environmental Determinants in Young people) is a population based longitudinal cohort study designed to investigate factors associated with diet and physical activity behaviour of children across the county of Norfolk, UK. The study's methods are described in detail elsewhere (van Sluijs et al., 2008, Corder et al., 2014) and so are only briefly recounted here.

In 2007, schools across Norfolk with at least 12 Year 5 pupils (age 9/10 years) were sampled according to stratification by urban/rural status (Bibby and Shepherd, 2004). Ninety two schools took part in the main study, and 2064 children were recruited. Baseline data collection was performed during the school summer term (April–July; ‘SPEEDY 1’). Teams of trained Research Assistants performed measurements at participating schools according to standard operating procedures. Participant height and weight were recorded using a Leicester height measure and non-segmental Tanita scales (type TBF-300A). Body mass index (BMI) was calculated from height and weight measurements and weight status (overweight or obese vs healthy weight) was determined based on international age and sex-specific cut points (Cole et al., 2000). Participants were fitted with an accelerometer (Actigraph GT1M) and were given a pack to take home including a questionnaire for their parents or carers to complete. To provide a measure of household socio-economic status, the parent questionnaire asked at what age the person completing the questionnaire (the mother on 84% of occasions) left full-time education.

Participants were invited to undertake further physical activity measurements in the school summer terms at +1year (2008), and again at +4years (2011) when aged 13/14 y and in Year 9, the third year of secondary education. At age 13–14 years the full suite of study measures (physical activity, diet, anthropometry and questionnaires) were repeated. For these analyses physical activity measurements from baseline and second (+4years) follow-up (‘SPEEDY 3’) were used, as these allow measurement of changes in behaviour between primary and secondary schools.

### 2.2 Physical activity measurement

The Actigraph GT1M accelerometers were set to record at 5 s epochs. Participants were asked to wear the devices on their right hip for seven days, removing them overnight and for aquatic activities. For consistency, and to limit any potential reactivity effect (Dössegger et al., 2014), the first partial day of data collection was removed from all files, and 10 min of continuous zero counts were classified as ‘non-wear time’ based on standard protocols (Eiberg et al., 2005, Mattocks et al., 2008, Riddoch et al., 2004). ‘Wear time’ was derived by subtracting minutes of ‘non-wear time’ from the total minutes in a given period. As physical activity outcomes were to be derived for two school-specific time periods, the commuting period (8–9 am and 3–4 pm), and the lunchtime period (12 noon to 2 pm), days for which fewer than 60 min of wear time were recorded within each of these two periods (across the two one-hour periods for commuting time) were excluded. Weekend days and school holidays were also excluded. Participants were included in the analysis if they provided at least one day of measurement on both measurement occasions, but were excluded if their baseline measurements were part of the pilot phase that was undertaken in February 2007. These criteria were implemented in order to maximize the numbers included in these analysis, and are in line with previous work with this sample (Corder et al., 2014, van Sluijs et al., 2008).

For each valid measurement day, time spent in MVPA (>2000 cpm) was extracted for the commuting period (8–9 am and 3–4 pm), and the lunchtime period (12 noon to 2 pm). MVPA during these times was averaged across all valid days at each measurement occasion for each participant. The threshold of 2000 cpm is equivalent to walking at 4 km/h (Ekelund et al., 2003) and has been used to define MVPA previously in this study (Corder et al., 2010, van Sluijs et al., 2008) and others (Riddoch et al., 2004). The outcome was average minutes of MVPA over each time period, and average wear time within the period was included as a covariate in all models. Change in average MVPA between the two time points was calculated by subtracting baseline average from follow-up average so that negative values indicate a decline in average time spent in MVPA.

### 2.3 School environment measurement

As part of the first phase of the SPEEDY study, we developed and tested an audit tool to objectively assess the opportunities for physical activity within primary school environments (Jones et al., 2010). The 44 item tool was used at the 92 primary schools recruited at baseline. Scores from the tool covering six domains of facility provision were examined against objectively measured time spent in MVPA among 1868 9–10 year old pupils attending the schools. The tool was found to have acceptable reliability and good construct validity, differentiating the physical activity levels of children attending the highest and lowest scoring schools (Jones et al., 2010).

For the +4year follow-up measurements at secondary schools, the SPEEDY school grounds audit was adapted very slightly from the original audit whereby three facilities that were commonly recorded as ‘other’ facilities in the original audit (‘formal garden/quiet space’, ‘outdoor teaching space’ and ‘vegetable/fruit garden’) were added as named items. No items were removed from the audit. Audit scores were calculated for SPEEDY 3 schools using the same methodology as for SPEEDY 1 (Jones et al., 2010). Briefly, these scores were derived by summing the values of individual items across six domains; ‘walking provision’, ‘cycling provision’, ‘sports and play provision’, ‘other facility provision’, ‘design of the school grounds’ and ‘aesthetics’. A seventh score was also created assessing overall school physical activity suitability by summing all the items included in the ‘walking provision’, ‘cycling provision’, ‘sports and play provision’ and ‘design of the school grounds’ domains.

In addition to the grounds audit, in order to provide a measure of each school's wider setting, the urban–rural location of each school was determined based on the lower super output area (LSOA; a unit of UK census geography containing 1000–3000 people) it fell within and the typology developed by Bibby and Shepard (2004).

### 2.4 Statistical analysis

Differences in audit scores between SPEEDY 1 and SPEEDY 3 were assessed using Wilcoxon rank-sum tests. Differences in the presence and absence of different facilities between the two measurement phases were assessed using Fisher's exact tests.

Cross-sectional associations between SPEEDY 3 audit scores and young people's MVPA were assessed using multilevel regression models, allowing for the clustering of young people within schools. The association between MVPA and each grounds audit score was assessed in separate models adjusting for sex, weight status, and accelerometer wear time over the relevant period. For the active travel related scores, household socio economic status (SES) based on the age the parent or guardian completing the baseline parental questionnaire reported that they left full time education, and home urban/rural location were also included as covariates. As their distributions and ranges varied considerably, all audit scores were banded into centile groups, with the number of categories dependent on the range of the score.

Outcome variables were average commuting time MVPA (8–9 am and 3–4 pm) for the ‘walking provision’ and ‘cycling provision’ scores, and average lunchtime MVPA (12 noon to 2 pm) for the ‘sports and play provision’, ‘other facility provision’, ‘design of the school grounds’ and ‘aesthetics’ scores. The overall school PA suitability score was tested with combined MVPA from commuting and lunch times. These models were initially fitted for all pupils, and then stratified by two pre-specified effect modifiers: sex and distance to school (commuting models only). Distance to school has previously been identified as a key determinant of active travel (Panter et al., 2008), and has also been seen to modify the association with its correlates (Panter et al., 2010). Stratification was based on the criterion distance for walking to school among SPEEDY 3 participants of 3 km (Chillón et al., 2014). Distance to school was the length of the shortest route between home and school address along a road network as determined using the ArcGIS Geographic Information System (ESRI Inc., 2012). In order to examine adjusted trends, the models were used to predict physical activity outcomes for each audit score centile at the mean values of other covariates.

The impact of the change in school environment supportiveness on change in MVPA as children move from primary to secondary schools was assessed in cross-classified multilevel regression models. The cross-classified component allowed for clustering of children within both primary and secondary schools, and provided estimates of residual variance at both these clusters. To aid interpretation, a simple model of change in school ground scores was adopted. Each primary and secondary school was classed as being more supportive or less supportive in terms of each audit score, with scores above and below the median respectively at each study phase. This resulted in four possible score change categories: less supportive at both schools, more supportive primary to less supportive secondary, less supportive primary to more supportive secondary and more supportive at both schools, with the latter category being used as the reference in the models. All models also included the same covariates as the cross-sectional models with wear-time expressed as the difference in accelerometer wear time over the same period (e.g. wear time during the lunchtime period for models of MVPA during lunchtime), and were run for all participants and stratified by sex. As both rounds of data collection were conducted over the summer term no adjustment was made for the effect of season.

To describe the proportion of residual variance at each level in the data hierarchy, variance partition coefficients (VPC) were calculated for null models, and for the final models with all explanatory variables added. All analyses were undertaken in Stata version 11 (StataCorp, 2009).

## 3 Results

### 3.1 Study participants

Of the 2064 participants originally recruited to the SPEEDY study, 480 consented to take part in SPEEDY 3. Of these 333 met the inclusion criteria for these analyses. Of the 147 who did not meet the inclusion criteria 52 consented to take part in the study, but provided no physical activity data, 62 had physical activity measurements taken during the summer holiday, and 33 did not provide at least 60 min of physical activity measurements during the commuting and lunchtime periods on at least one school day at both measurement occasions.

The SPEEDY 3 grounds audit was conducted at 47 school sites. This included two schools with split sites for which separate audits were conducted at each site. These four audits were included in the comparison of primary and secondary school audits, but were not linked to participants’ physical activity measures as we did not know which site young people would have used and when. Participants attending these secondary schools were also excluded from these analyses leaving a final sample of 299 participants at 43 secondary schools for the analyses of physical activity. Of the 299 participants, 244 provided ≥3 days of data at both measurement occasions. Of the 55 participants who provided <3 days at one measurement occasion, 49 provided ≥3 days at the other measurement occasion.

Table 1 shows the characteristics of the participants included in these analyses. As previously reported (Corder et al., 2014), MVPA declined between SPEEDY 1 and SPEEDY 3 during all periods of the school day except commuting times. Based on SPEEDY 1 measurements, those included in these analysis show some statistically significant differences to those excluded (*p*<0.05). Those included tended to have lower BMI (mean=17.95 among those included vs 18.30 among those excluded), be of a higher SES (58.82% had a parent finishing full time education aged 16+ vs 50.55% among the excluded), and register more accelerometer wear time during the commuting period than those not included (mean=109.8 min vs 111.0).

### 3.2 Change in school environments between primary and secondary schools

Table 2 shows a summary of school audit scores for SPEEDY 1 (primary) and SPEEDY 3 (secondary) schools. While secondary schools generally scored lower in the composite scores, significantly so for sport and play facilities, design of the school grounds and the overall physical activity suitability score, this hides the changes to some of the individual components underlying these scores (shown in Supplementary Table 1). In terms of statistically significant differences (*p*<0.05), the secondary schools we audited were less likely to have traffic calming features such as speed bumps near their entrances, but were more likely to have cycle lanes and cycle route signs. The secondary schools had fewer brightly coloured markings on playgrounds, and fewer pieces of fixed play equipment, but more sports pitches, courts and athletics tracks. They were also significantly less likely to have a hard-surface playground (73% had them, compared to 97% of the primary schools), but were more likely to be rated as very suitable for sports.

### 3.3 Cross-sectional associations between the secondary school environment and young people's physical activity

Table 3 shows the cross-sectional association between school-based MVPA and grounds audit scores for secondary schools. During commuting time, there was a significant trend of increases in the amount of MVPA recorded at schools with higher scores for both walking and cycling provision. Overall, students at the highest scoring schools for cycling provision acquired an average of almost six minutes more MVPA than those at the lowest scoring schools. When stratified by sex, this pattern was similar for girls and boys, but only reached statistical significance among boys.

There were no significant differences in lunchtime MVPA at schools with differing sport and play facilities, other facilities, design or aesthetics scores. Similarly no association was seen between MVPA during commuting and lunchtime and the overall school physical activity suitability score.

Stratifying the analysis of walk and cycle provision scores by distance to school (Table 4) showed that the association seen among all participants was being driven by those living further from school. It was among these young people that a statistically significant association existed between commuting time MVPA walking and cycling provision scores. The association was not seen in those living nearer to school.

### 3.4 Longitudinal association between change in children's physical activity and change in school environment

Multilevel regression models predicting change in MVPA by change in school supportiveness score between SPEEDY 1 and SPEEDY 3 are shown in Table 5. We found no association between MVPA change and school audit score change in all but two cases. Among boys, moving from a less supportive primary school to a less supportive secondary school in terms of sport and play facilities provision (mean change 0.91 min, 95% CI −3.06, 4.89, *p*=0.008) and overall school suitability (mean change 7.03 min, 95% CI −0.57, 14.63, *p*=0.002) was associated with an increase in MVPA at lunch times and at lunchtime plus commuting times respectively.

Table 6 shows variance partition coefficients (VPC) for the unconditional (null) and conditional models of commuting and lunchtime MVPA by change in overall school PA suitability score. The VPCs indicate the proportion of the unexplained variation that was attributable to the different levels of the model hierarchy (pupil, primary school and secondary school). In the unconditional models, when looking at the model for both sexes combined, around 7% of the variation in change in MVPA lay between both primary and secondary schools. These values were different when stratified by sex, so that for girls a greater proportion of the variation in change in MVPA lay at the primary school (6.7%), whereas for boys more variation sat between secondary schools (19.0%). VPCs changed when explanatory variables were added in the conditional models. Overall, as expected, the unexplained variance in change in MVPA was reduced, but a much larger percentage remained at secondary school level, suggesting that unmeasured factors at secondary schools may have played some part in driving differences in observed changes in MVPA.

## 4 Discussion

We found differences in the environmental supportiveness of school grounds for physical activity between primary and secondary schools in the SPEEDY study. However, cross-sectional associations between secondary school supportiveness and school based MVPA were only seen for active travel provision scores and commuting time MVPA. Very few significant associations were seen between change in school supportiveness and change in school-based MVPA across the transition from primary to secondary school and those that were observed were in a counterintuitive direction.

The three aims of this paper were formulated in order to better understand the physical environments of secondary schools, and how their characteristics may be associated with young people's physical activity. In assessing the first aim, comparisons of the scores derived from the SPEEDY school grounds audit revealed some considerable differences between primary school and secondary school environments. While primary schools are relatively well studied settings with regard to their supportiveness for physical activity, secondary schools have received much less attention. The differences we saw between the school types suggest that they constitute quite different physical settings, possibly requiring different adaptive strategies.

Previous analysis of the SPEEDY 1 data showed positive cross-sectional associations between school based MVPA and walking and cycling provision scores, and the provision of sport and play facilities, and other facilities (Jones et al., 2010). However, it was only the walking and cycling provision scores that were significantly associated with commuting time MVPA at SPEEDY 3. Stratification by pupil distance to school showed that these associations were mainly driven by those living further from school. The associations were not seen in those living nearer to school, suggesting that for those who live near enough, school characteristics are unimportant as determinants of travel mode, but for those who live further away, the additional support for active travel given by schools who scored more highly on these domains may be beneficial in increasing young people’s commuting time MVPA.

The SPEEDY grounds audit tool was developed for use in primary school settings, where its reliability and validity have previously been tested. As the audit tool used among the secondary schools and its accompanying user manual remain almost entirely unchanged from the primary school version there is no reason to think that its inter-operator reliability would have changed. However, we cannot necessarily assume that its construct validity is the same among secondary schools, although past studies have used the same environmental measurement tools in both settings (De Meester et al., 2014, Haug et al., 2010). In addressing our second aim we did not see any associations between the non-active travel audit scores and young people’s school based MVPA. This could be because items deemed relevant for physical activity in primary school grounds are not relevant in secondary school settings. Future work assessing the physical environment of secondary schools should look to refine and modify existing primary school based tools, and sample from as wide a range of settings as possible.

Although cross-sectional associations were seen between walking and cycling scores and commuting time physical activity at SPEEDY 1 and SPEEDY 3, we did not see a longitudinal association between changes in these scores and changes in commuting time MVPA. The geography of our study setting and the organization of schools in England mean that many of our participants attended small, village primary schools, moving to larger, more urban secondary schools much further from home. The impact of this on participants’ travel mode was a large increase in the proportion of children travelling to school by bus (6.2% at SPEEDY 1, 35.8% at SPEEDY 3 (Chillón et al., 2014)), and a reduction in the numbers using active modes (48.8% at SPEEDY 1, and 38.9% at SPEEDY 3 (Chillón et al., 2014)). These large changes in modes highlight the presence of much stronger influences on change in children’s commuting time physical activity than school ground supportiveness.

For the non-active travel related audit scores we saw very few significant associations between change in school supportiveness and change in MVPA, and those we did see were not in the direction we expected. The lack of associations seen may be due to differences in what constitutes supportive school grounds in primary and secondary school settings. We saw considerable differences in the sport and play facilities recorded at secondary schools compared to primary schools. Very few bright markings on play surfaces or playground equipment such swings or slides were recorded at secondary schools, whereas they had much greater numbers of sports pitches and courts. While these types of facilities hypothetically provide space and structure for school-based physical activity, and are often cited as important facilities within secondary schools (De Meester et al., 2014, Haug et al., 2010, Haug et al., 2008), it is possible that they are not as well used during lunch time in secondary schools as playground markings and equipment are in primary schools (Willenberg et al., 2010). They may therefore be not as supportive of young people’s physical activity, which could also explain the lack of a cross-sectional association at SPEEDY 3.

De Meesters et al. (2014) found that secondary schools in their Belgian sample tended to score higher than primary schools in terms of numbers of physical activity facilities and pieces of equipment, whereas we found that secondary schools scored lower on average for sport and play facilities provision. Also in contrast to our findings, they saw that increases in facilities and equipment were associated with increases in pedometer-measured weekday step-counts, although not accelerometer determined MVPA (De Meester et al., 2014). Unlike the questionnaire used by De Meesters et al. (2014), which obtained measures of the school grounds as perceived by individual head teachers, our audit objectively assessed several domains of school ground physical activity supportiveness. These covered not only the presence of specific grounds components, but also objective assessments of active commuting facilities and local road safety features, and design elements of the grounds (e.g. topography, and suitability for unstructured play), which may prove important in terms of providing guidance for the how to build or alter school grounds to optimize physical activity levels among students.

A recent paper by Marks et al. (2015) found that physical activity declines were greater among children who changed schools at the primary/secondary transition than those who remained in the same school (the Australian system in which their work was based includes some combined primary-secondary schools). Their finding highlights the importance of a change in school environment to physical activity levels, but our results do not support the notion that these changes are purely a result of the physical change in environment.

It is possible that the timing of young people's MVPA differs as they age, and that the school lunch period becomes a less important time for physical activity, especially as lunch breaks tend to be shorter in secondary schools. Past work on the SPEEDY study found that MVPA declined more steeply out of school and at lunch times compared with during lesson-times (Brooke et al., in press) and in this sample a smaller proportion of participants' school time MVPA was undertaken during the lunch period at follow-up than at baseline (mean 51.7% vs 44.3%, p for difference <0.01). However fitting MVPA accrued throughout the whole school day (9 am to 3 pm) as the outcome in our models did not produce results substantively different to those presented.

The VPCs from our models show that a significant portion of the unexplained variance in physical activity change sits at the secondary school level, suggesting that there are other secondary school level factors that are important for change in school-based MVPA, but that were not captured in the audit. These could include other elements of the grounds that were not included in the audit, or the policy environment or wider physical and social contexts of the schools.

Many factors shape children's physical activity habits as they age, and although schools are seen as important settings in which to be active, the nature of the grounds themselves are not necessarily the most important features. Policies around the space and time young people have for physical activity change between primary and secondary schools, although break times are consistently seen as opportunities for a wide range of activities, both sedentary and active (Gorely et al., 2011). However, it is likely to be a combination of social, cultural, and physical environmental factors that are most effective in promoting physical activity in school. Changes to school policy must be backed up by a supportive physical environment to maximize their effectiveness (Doak et al., 2006), and similarly changes in the physical environment must coincide with policy changes in order for them to be effective at a population level (Morton et al., in press).

This study has some strengths and limitations. We used a validated, objective measure of physical activity, and followed up the same children in order to assess change in behaviour in relation to change in environment. Although for simplicity we chose to model only one physical activity outcome (MVPA), we also ran our models using counts per minute (cpm) and sedentary time (defined as <100 cpm) as outcomes, obtaining very similar results (results not shown, associations in the opposite direction for sedentary time). This suggests that our findings are not strongly dependent on the threshold used to determine MVPA. We also used an existing audit tool, the reliability and validity of which has previously been tested in a primary school setting, to objectively assess the suitability for physical activity of a large number of schools.

Limitations must also be acknowledged. The sample included in these analyses represented 15% of the original SPEEDY sample, which itself contained an higher proportion of girls, and a lower proportion of obese children than the Norfolk population (van Sluijs et al., 2008). Considering baseline measurements, those included in these analyses had lower BMI, and were of higher SES, than those not included, possibly reducing the generalizability of these findings. In addition, the Norfolk population, and hence our sample, is largely white, potentially making these results less relevant to other populations.

Our audit could only capture the presence of fixed pieces of equipment such as goal posts, swings, slides, and not smaller, portable equipment such as balls, bats and Frisbees, the presence of which has previously been associated with increased MVPA in secondary school students (Ridgers et al., 2013). It is possible that schools that lack sport and play facilities and equipment within their grounds are more likely to provide this type of play equipment. We were also unable to account for school policy decisions which may impact how and when students access the grounds and are allowed to use their facilities.

To conclude, we saw considerable differences in the nature of school grounds between primary and secondary schools in Norfolk, UK. Cross-sectional associations were seen between walking and cycling provision scores and secondary school student’s commuting time MVPA, which appeared to be particularly relevant for boys and adolescents living further from school. These results suggest that improving active travel facilities at schools where pupils tend to live further away may be beneficial for their physical activity. However, despite the secondary school environment appearing to explain a substantial amount of variance in change in activity, few associations were seen between changes in school grounds scores and changes in school-based physical activity, providing no indications for the design or likely efficacy of school-grounds based physical activity interventions in secondary schools. Future research may want to consider the internal environment of schools, loose equipment provision, and the policy and social environments of schools, and in particular how these interact with the physical environment, in order to further understand the best ways of preventing the decline in physical activity across the transition to adolescence.

## Authors contributions

FH formulated the research question, carried out the analyses and drafted the initial manuscript. KC supervised and coordinated follow-up data collection. AJ and EMFvS were involved with the conceptualization and design of the initial SPEEDY study and supervision of data collection. All authors critically reviewed the manuscript, and approved the final manuscript as submitted.

## Funding

The SPEEDY study is funded by the National Prevention Research Initiative (code G0501294) (http://www.npri.org.uk), consisting of the following Funding Partners: British Heart Foundation; Cancer Research UK; Department of Health; Diabetes UK; Economic and Social Research Council; Medical Research Council; Health and Social Care Research and Development Office for the Northern Ireland; Chief Scientist Office, Scottish Government Health Directorates; Welsh Assembly Government and World Cancer Research Fund. This work was also supported by the Medical Research Council (Unit Programme numbers MC_UU_12015/7, MC_UU_12015/4, and MC_UU_12015/3) and the Centre for Diet and Activity Research (CEDAR), a UKCRC Public Health Research: Centre of Excellence. Funding from the British Heart Foundation, Economic and Social Research Council, Medical Research Council, the National Institute for Health Research, and the Wellcome Trust, under the auspices of the UK Clinical Research Collaboration, is gratefully acknowledged (Grant code RES-590-28-002).

Ethics approval: University of East Anglia Ethics Committee.

## Figures and Tables

**Table 1:** Characteristics of SPEEDY participants included in these analyses.

	Number (%) or mean (standard deviation)		
	9–10 years (2007)	13–14 years (2011)	
Sex (Female)	165 (55.2%)	–	
Age	10.2 (*0.3*)	14.3 (*0.3*)	
BMI*	18.0 (*3.1*)	20.8 (*4.0*)	
Weight status^a^			
Healthy weight	241 (80.9%)	240 (80.8%)	
Overweight	47 (15.8%)	44 (14.8%)	
Obese	10 (3.4%)	13 (4.4%)	
Age parent left full time education^b^^,^*			
≤16	119 (41.2%)	–	
16–18	104 (36%)	–	
>18	66 (22.8%)	–	
Home location			
Urban	120 (40.1%)	116 (38.8%)	
Town & Fringe	68 (22.7%)	65 (21.7%)	
Rural	111 (37.1%)	118 (39.5%)	
Average time spent (Lunchtime 12 noon to 2 pm)…			
MVPA	14.5 (*5.8*)	10.9 (*5.7*)	†
Registered time	116.4 (*5.2*)	112.6 (*8.9*)	†
Average time spent (Commuting time 8−9 am and 3−4 pm)…			
MVPA	15.9 (*7.1*)	17.9 (*11.3*)	†
Registered time*	111 (*9.4*)	105.1 (*11.5*)	†
Average time spent (School day 9 am to 3 pm)…			
MVPA	28.2 (*9.9*)	24.4 (*10.2*)	†
Registered time	342.7 (*17.3*)	323.0 (*27.2*)	†
Average time spent (After school 4–9 pm)…			
MVPA	28.9 (*14.2*)	19.8 (*13.5*)	†
Registered time	254.0 (*27.4*)	251.8 (*30.9*)	

a Weight status missing for 1 participant at baseline and 2 participants at follow-up.

b Age parent left full time education missing for 10 participants.

* At SPEEDY 1 those included in analysis significantly different (*p*<0.05) to those not included.

† For physical activity measures, differences between baseline and follow-up statistically significant (*p*<0.05).

**Table 2:** Summary of school physical activity supportiveness grounds audit at primary and secondary schools.

	Mean (standard deviation)		
	Primary schools (*N*=92)	Secondary schools (*N*=43)	*p* For difference
Cycle provision score	3.53 (1.38)	3.78 (1.68)	0.182
Walking provision score	2.13 (0.76)	2.33 (0.88)	0.083
Sport and play facilities score	8.10 (1.87)	6.09 (1.66)	**<0.001**
Other facilities score	3.54 (1.57)	3.36 (2.14)	0.281
Design of the school grounds score	9.16 (0.81)	6.33 (1.09)	**<0.001**
Aesthetics score	21.69 (2.26)	21.80 (2.65)	0.403
Overall physical activity suitability	19.69 (2.43)	14.51 (2.47)	**<0.001**

**Table 3:** Cross-sectional associations between secondary school-based MVPA and audit score percentiles, in the whole sample and stratified by sex.

		All (*N*=301)		Girls (*N*=165)		Boys (*N*=136)	
		mean^a^ MVPA (SE)	*p*^b^	mean^a^ MVPA (SE)	*p*^b^	mean a MVPA (SE)	*p*^b^
Commuting time MVPA (8–9 am and 3–4 pm)							
Walking provision							
	Lowest tertile	17.1 (2.5)		20.3 (3.1)		13.8 (2.8)	
		19.1 (1.9)		21.7 (2.2)		17.1 (2.1)	
	Highest tertile	22.2 (1.8)	**0.036**	26 (1.9)	0.098	19.9 (1.9)	**0.028**
Cycling provision							
	Lowest quartile	17.2 (2.6)		20.3 (3)		14.2 (2.9)	
		19.0 (1.8)		22.0 (2.2)		16.7 (1.9)	
	Highest quartile	20.1 (3.0)		21.7 (3.3)		19.2 (2.8)	
Lunchtime MVPA (12 noon to 2 pm)		23.3 (2.0)	**0.022**	26.8 (2.1)	0.084	21.2 (2.2)	**0.017**
Sport and play facilities							
	Lowest quartile	13.1 (0.9)		9.0 (0.9)		12.8 (1.3)	
		14.8 (1.2)		8.7 (1.2)		17.1 (1.7)	
		13.1 (0.8)		8.5 (0.8)		13.5 (1.2)	
	Highest quartile	13.9 (0.8)	0.849	9.7 (0.8)	0.797	14.5 (1.1)	0.657
Other facilities provision							
	Lowest quartile	13.4 (0.9)		9.2 (0.9)		13.4 (1.4)	
		14.4 (1.1)		9.8 (1.0)		14.6 (1.6)	
		13.1 (0.7)		8.4 (0.6)		13.7 (1.1)	
	Highest quartile	14.1 (0.9)	0.715	9.4 (1.1)	0.323	15.3 (1.4)	0.674
Design of the school grounds							
	Lowest tertile	13.7 (1.2)		9.9 (1.0)		13.2 (2.1)	
		13.1 (0.9)		8.3 (0.8)		13.8 (1.3)	
	Highest tertile	13.7 (0.6)	0.802	9.1 (0.7)	0.550	14.4 (0.9)	0.546
Aesthetics							
	Lowest quintile	14.6 (0.8)		10.0 (0.7)		15.1 (1.3)	
		12.9 (1.3)		8.4 (1.2)		12.8 (2.3)	
		13.5 (0.9)		8.8 (0.8)		13.9 (1.5)	
		12.6 (0.7)		7.4 (0.6)		13.5 (1.2)	
	Highest quintile	14.7 (1.0)	0.462	11.6 (1.1)	0.390	14.7 (1.6)	0.711
Commuting and lunch times combined							
Overall school PA suitability							
	Lowest quintile	30.2 (3.4)		30.9 (4.5)		26.0 (3.9)	
		34.1 (3.0)		30.8 (3.2)		34.8 (3.5)	
		32.9 (3.0)		30.9 (3.2)		32.3 (3.6)	
		34.6 (2.6)		34.0 (2.6)		31.4 (3.1)	
	Highest quintile	34.1 (2.5)	0.393	34.9 (2.6)	0.282	31.4 (2.9)	0.598

a Adjusted for sex (for ‘All’ summary), weight status (overweight/obese vs healthy weight), registered time, age parent left full-time education, and home urban/rural location.

b *p* Value for test for trend across percentile groups.

**Table 4:** Associations between commuting time MVPA and audit walk and cycle score percentiles, stratified by distance to school.

					Near (*N*=138)		Far (*N*=163)	
					Mean^a^ MVPA (SE)	*p*^b^	Mean^a^ MVPA (SE)	*p*^b^
Commuting time MVPA (8–9 am and 3–4 pm)								
Walking provision								
			Lowest tertile		24.7 (5.0)		12.2 (2.1)	
					24.8 (3.1)		14.0 (1.8)	
			Highest tertile		23.7 (2.3)	0.737	16.3 (1.8)	**0.036**
Cycling provision								
			Lowest quartile		24.7 (4.8)		11.9 (2.1)	
					26.4 (2.9)		13.3 (1.6)	
					19.9 (3.5)		14.2 (2.3)	
			Highest quartile		24.1 (2.6)	0.650	18.1 (1.9)	**0.003**

a Adjusted for sex, weight status (overweight/obese vs healthy weight), registered time, age parent left full-time education, and home urban/rural location.

b *p* Value for test for trend across centile groups.

**Table 5:** Models predicting change in MVPA by change in school ground audit scores.

	All (*N*=299)				Girls (*N*=165)				Boys (*N*=134)			
	Mean change in MVPA^a^	95% CI	*p*^b^	*N*	Mean change in MVPA^a^	95% CI	*p*^b^	*N*	Mean change in MVPA^a^	95% CI	*p*^b^	*N*
Commuting time												
Walk score change^c^												
More to more	1.59	−0.30, 3.49		234	3.46	1.21, 5.70		133	0.32	−1.81, 2.45		101
Less to less	−0.88	−13.94, 12.18	*0.710*	2	3.26	−16.19, 22.72	*0.984*	1	−5.93	−22.3, 10.43	*0.454*	1
More to less	−1.10	−5.66, 3.47	*0.249*	40	−0.17	−6.03, 5.7	*0.226*	20	−2.56	−7.4, 2.28	*0.244*	20
Less to more	0.14	−4.00, 4.29	*0.493*	23	3.98	−2.61, 10.57	*0.877*	11	−2.03	−7.35, 3.29	*0.386*	12
Cycle score change^c^												
More to more	1.51	−0.41, 3.43		232	3.42	1.10, 5.73		129	−0.04	−2.11, 2.04		103
Less to less	1.44	−7.06, 9.94	*0.987*	6	5.41	−7.64, 18.46	*0.765*	3	−2.82	−13.17, 7.53	*0.598*	3
More to less	−1.34	−6.26, 3.59	*0.257*	33	−0.03	−6.29, 6.23	*0.280*	18	−2.71	−8.00, 2.57	*0.320*	15
Less to more	0.39	−3.63, 4.42	*0.586*	28	2.83	−3.13, 8.79	*0.848*	15	0.26	−5.10, 5.63	*0.913*	13
Lunchtime												
Sport and play facilities change^c^												
More to more	−4.17	−5.35, −2.98		110	−3.96	−5.16, −2.76		65	−4.45	−6.45, −2.44		45
Less to less	−2.06	−4.38, 0.25	*0.075*	36	−3.63	−5.92, −1.33	*0.777*	21	0.91	−3.06, 4.89	**0.008**	15
More to less	−4.54	−6.55, −2.53	*0.717*	69	−3.18	−5.30, −1.06	*0.470*	35	-5.70	−8.86, −2.54	*0.436*	34
Less to more	−2.76	−4.35, −1.18	*0.082*	84	−2.82	−4.49, −1.14	*0.182*	44	−3.03	−5.76, −0.3	*0.308*	40
Other Facilities change^c^												
More to more	−4.06	−5.10, −3.01		123	−4.09	−5.14, −3.03		74	−4.07	−5.99, −2.16		49
Less to less	−5.12	−7.14, −3.09	*0.305*	63	−3.94	−6.01, −1.86	*0.886*	35	−6.44	−10.11, −2.77	*0.206*	28
More to less	−2.20	−4.00, −0.41	0.043	68	−2.08	−3.95, −0.22	0.035	38	−2.60	−5.66, 0.47	*0.345*	30
Less to more	−3.05	−4.76, −1.33	*0.247*	45	−2.93	−4.92, −0.93	*0.253*	18	−2.34	−5.27, 0.59	*0.245*	27
Design of grounds change^c^												
More to more	−3.67	−4.78, −2.56		23	−3.58	−4.74, −2.42		18	−3.94	−5.67, −2.21		5
Less to less	−2.56	−5.43, 0.31	*0.447*	19	−3.97	−6.57, −1.37	*0.768*	13	1.34	−4.11, 6.80	*0.058*	6
More to less	−3.82	−5.52, −2.13	*0.865*	109	−3.22	−4.99, −1.46	*0.691*	62	−4.46	−7.17, −1.75	*0.707*	47
Less to more	−2.83	−5.19, −0.46	*0.482*	148	−3.35	−5.47, −1.23	*0.830*	72	−1.26	−6.10, 3.58	*0.278*	76
Aesthetics change^c^												
More to more	−3.27	−4.73, −1.81		67	-3.70	−5.22, −2.19		33	−2.92	−5.35, −0.49		34
Less to less	−2.99	−5.02, −0.96	*0.788*	105	−2.58	−4.61, −0.54	*0.279*	63	−3.53	−6.99, −0.06	*0.732*	42
More to less	−3.58	−5.68, −1.49	*0.770*	64	−3.53	−5.78, −1.28	*0.880*	31	−3.63	−7.04, −0.21	*0.685*	33
Less to more	−4.45	−6.41, −2.50	*0.237*	63	−4.16	−6.08, −2.24	*0.640*	38	−4.98	−8.44, −1.51	*0.245*	25
Commuting and lunch times												
Overall school suitability change^c^												
More to more	−1.88	−4.71, 0.95		129	0.63	−2.61, 3.87		83	−5.23	−8.89, −1.58		46
Less to less	−0.02	−5.44, 5.40	*0.501*	32	−1.97	−8.45, 4.52	*0.433*	22	7.03	−0.57, 14.63	**0.002**	10
More to less	−4.91	−9.21, −0.62	*0.166*	91	−2.42	−7.73, 2.90	*0.261*	42	−7.08	−12.21, −1.95	*0.480*	49
Less to more	−2.13	−6.22, 1.96	*0.905*	47	1.05	−5.42, 7.52	*0.899*	18	−3.98	−8.94, 0.97	*0.621*	29

a All adjusted for change in registered time, baseline MVPA, sex (for ‘All’ summary), and weight status (overweight/obese vs healthy weight), commuting time and combined commuting and lunchtime models also adjusted for household SES and home urban/rural location.

b *p* For difference to reference category (more supportive to more supportive). *p* values are therefore not given for mean change in MVPA in the reference category.

c For all models, reference category is moving from a more supportive primary school to a more supportive secondary school.

**Table 6:** Random effects parameters from models predicting change in commuting and lunchtime MVPA by change in overall school grounds audit score.

	All				Girls				Boys			
		95% CI				95% CI				95% CI		
	Variance	Lower	Upper	VPC (%)	Variance	Lower	Upper	VPC (%)	Variance	Lower	Upper	VPC (%)
Unconditional models												
Secondary School	12.0	2.8	52.6	6.4	3.6	0.0	18790.2	1.7	18.6	4.3	80.4	11.7
Primary School	13.5	3.1	58.2	7.1	21.0	3.7	120.4	10.1	15.0	1.7	133.3	9.4
Pupil	164.0	138.1	194.8	86.6	183.9	145.4	232.4	88.2	125.0	92.9	168.1	78.8
Total	189.5				208.4				158.6			
Conditional models^a^												
Secondary School	20.7	9.8	43.9	16.7	18.2	3.7	90.6	14.5	19.0	7.0	51.6	18.5
Primary School	0.0	0.0	0.0	0.0	6.7	0.2	287.8	5.4	0.7	0.0	8.1E+09	0. 7
Pupil	103.5	87.3	122.7	83.3	100.7	77.8	130.2	80.1	83.2	62.1	111.3	80.9
Total	124.2				125.6				102.8			

a Models include the variables: change in overall school grounds supportiveness, change in registered time, baseline MVPA, sex, weight status at follow-up, age parent left full-time education, and home urban/rural location.
