# Supplementary material for: School grounds and physical activity: Associations at secondary schools, and over the transition from primary to secondary schools
Source: Health Place. 2016 May;39:34–42. doi: 10.1016/j.healthplace.2016.02.004 (PMC5405048; doi:10.1016/j.healthplace.2016.02.004)
Supplement: Supplementary file 1 — Supplementary material [file mmc1.doc]

Supplementary Table 1. Summary of individual items in grounds audits at primary and secondary schools.

|  | Summary statistic used ^a^ | Primary Schools (N=92) | Secondary Schools (N=43) | p |
| --- | --- | --- | --- | --- |
| Number of schools |  | 92 | 47 |  |
| Are the following visible from any school entrance? |  |  |  |  |
| A place where parents can stop and drop children off | N(%) yes | 90 (97.83%) | 43 (95.56%) | 0.598 |
| Places where parents can park their cars | N(%) yes | 87 (94.57%) | 38 (84.44%) | 0.175 |
| A bus stop | N(%) yes | 25 (27.17%) | 21 (46.67%) | **0.021** |
| Cycle lanes separate from the road | N(%) yes | 8 (8.7%) | 12 (26.67%) | **0.009** |
| Cycle lanes on the road | N(%) yes | 2 (2.17%) | 2 (4.44%) | 0.592 |
| Pavements on both sides of the road | N(%) yes | 57 (61.96%) | 38 (84.44%) | **0.005** |
| Pavements on one side of the road only | N(%) yes | 47 (51.09%) | 10 (22.22%) | **0.008** |
| A marked pedestrian crossing | N(%) yes | 4 (4.35%) | 8 (17.78%) | **0.019** |
| Traffic calming | N(%) yes | 69 (75%) | 23 (51.11%) | **0.011** |
| School warning signs for road users | N(%) yes | 79 (85.87%) | 34 (75.56%) | 0.327 |
| Road safety signs | N(%) yes | 3 (3.26%) | 1 (2.22%) | 1.000 |
| Route signs for cyclists | N(%) yes | 7 (7.61%) | 10 (22.22%) | **0.023** |
| Facilities within school grounds; numbers present of… | |  |  |  |
| Bright markings on play surfaces | med (IQR) | 6 (4 - 10) | 0 (0 - 0) | **<0.001** |
| Playground equipment (e.g. swings, slide) | med (IQR) | 0 (0 - 2) | 0 (0 - 0) | **<0.001** |
| Pitches (e.g. football, softball, cricket) | med (IQR) | 1 (0.5 - 2) | 4 (3 - 6) | **<0.001** |
| Athletics track (grass or hard-surface) | med (IQR) | 1 (0 - 1) | 1 (1 - 2) | **<0.001** |
| Courts (e.g. tennis, basketball, netball) | med (IQR) | 2 (1 - 4) | 9 (6 - 11) | **<0.001** |
| Benches | med (IQR) | 5 (2 - 8) | 6 (3 - 18) | 0.131 |
| Picnic tables | med (IQR) | 4 (3 - 6) | 8 (4 - 14) | **<0.001** |
| Drinking fountains | med (IQR) | 0 (0 - 0) | 0 (0 - 0) | 0.530 |
| A wildlife garden | med (IQR) | 0 (0 - 1) | 0 (0 - 1) | 0.189 |
| Uncovered cycle parking (number of bikes) | med (IQR) | 0 (0 - 10) | 0 (0 - 10) | 0.857 |
| Covered cycle parking (number of bikes) | med (IQR) | 10 (0 - 20) | 52 (27 - 120) | **<0.001** |
| Total cycle parking spaces | med (IQR) | 20 (10-30) | 60 (36-120) | **<0.001** |
| An assault course | med (IQR) | 1 (0 - 1) | 0 (0 - 0) | **<0.001** |
| Is dog mess visible in the areas where pupils play? | N(%) yes | 1 (1.09%) | 1 (2.22%) | 0.537 |
| Are the school grounds on a split site? | N(%) yes | 5 (5.43%) | 4 (8.89%) | 0.475 |
| Are the school grounds mainly flat or sloping? | N(%) Sloping | 12 (13.04%) | 5 (11.11%) | 1.000 |
| Does the school have a hard surface playground? | N(%) yes | 89 (96.74%) | 33 (73.33%) | **<0.001** |
| To what extent are the following are present? |  |  |  |  |
| Planted beds containing flowers/shrubs/etc | N = None (%) | 16 (17.39%) | 4 (8.89%) | 0.246 |
|  | N = Some (%) | 46 (50%) | 29 (64.44%) |  |
|  | N = A lot (%) | 30 (32.61%) | 12 (26.67%) |  |
| Trees for shade | N = None (%) | 12 (13.04%) | 4 (8.89%) | 0.229 |
|  | N = Some (%) | 51 (55.43%) | 20 (44.44%) |  |
|  | N = A lot (%) | 29 (31.52%) | 21 (46.67%) |  |

Supplementary Table 1. Cont... Summary of individual items in grounds audits at primary and secondary schools.

|  | Summary statistic used ^a^ | Primary Schools (N=92) | Secondary Schools (N=43) | p |
| --- | --- | --- | --- | --- |
| To what extent are the following are present? | |  |  |  |
| Loud ambient noise (e.g. traffic, trains, industry) | N = None (%) | 46 (50%) | 19 (42.22%) | 0.666 |
|  | N = Some (%) | 40 (43.48%) | 22 (48.89%) |  |
|  | N = A lot (%) | 6 (6.52%) | 4 (8.89%) |  |
| Litter | N = None (%) | 78 (84.78%) | 20 (44.44%) | **<0.001** |
|  | N = Some (%) | 12 (13.04%) | 17 (37.78%) |  |
|  | N = A lot (%) | 2 (2.17%) | 8 (17.78%) |  |
| Murals / outdoor art | N = None (%) | 66 (71.74%) | 31 (68.89%) | 0.803 |
|  | N = Some (%) | 16 (17.39%) | 10 (22.22%) |  |
|  | N = A lot (%) | 10 (10.87%) | 4 (8.89%) |  |
| Graffiti | N = None (%) | 92 (100%) | 41 (91.11%) | **0.011** |
|  | N = Some (%) | 0 (0%) | 3 (6.67%) |  |
|  | N = A lot (%) | 0 (0%) | 1 (2.22%) |  |
| Are the school grounds generally suitable for … ? | |  |  |  |
| Sport (organised or not)? | N = Not at all (%) | 1 (1.09%) | 1 (2.22%) | **0.001** |
|  | N = Somewhat (%) | 43 (46.74%) | 9 (20%) |  |
|  | N = Very (%) | 48 (52.17%) | 35 (77.78%) |  |
| Informal games? | N = Not at all (%) | 0 (0%) | 1 (2.22%) | 0.281 |
|  | N = Somewhat (%) | 9 (9.78%) | 6 (13.33%) |  |
|  | N = Very (%) | 83 (90.22%) | 38 (84.44%) |  |
| General play? | N = Not at all (%) | 0 (0%) | 1 (2.22%) | 0.136 |
|  | N = Somewhat (%) | 6 (6.52%) | 6 (13.33%) |  |
|  | N = Very (%) | 86 (93.48%) | 38 (84.44%) |  |
| To what extent do you agree or disagree with the following statements? | | |  |  |
| The grounds are shielded from the surrounding area by hedges/trees/fences | N(%) S disagree | 5 (5.43%) | 2 (4.44%) | **<0.001** |
|  | N(%) Disagree | 17 (18.48%) | 1 (2.22%) |  |
|  | N(%) Neither | 25 (27.17%) | 3 (6.67%) |  |
|  | N(%) Agree | 34 (36.96%) | 18 (40%) |  |
|  | N(%) S agree | 11 (11.96%) | 21 (46.67%) |  |
| The grounds are generally well maintained | N(%) S disagree | 0 (0%) | 0 (0%) | 0.190 |
|  | N(%) Disagree | 1 (1.09%) | 2 (4.44%) |  |
|  | N(%) Neither | 3 (3.26%) | 4 (8.89%) |  |
|  | N(%) Agree | 44 (47.83%) | 16 (35.56%) |  |
|  | N(%) S agree | 44 (47.83%) | 23 (51.11%) |  |
| The grounds are generally free of vandalism | N(%) S disagree | 0 (0%) | 0 (0%) | 0.208 |
|  | N(%) Disagree | 0 (0%) | 0 (0%) |  |
|  | N(%) Neither | 0 (0%) | 0 (0%) |  |
|  | N(%) Agree | 6 (6.52%) | 6 (13.33%) |  |
|  | N(%) S agree | 86 (93.48%) | 39 (86.67%) |  |

Supplementary Table 1. Cont... Summary of individual items in grounds audits at primary and secondary schools.

|  | Summary statistic used ^a^ | Primary Schools (N=92) | Secondary Schools (N=43) | Summary statistic used ^a^ |
| --- | --- | --- | --- | --- |
| Estimate the percentage cover of the following surfaces where children could play: | | |  |  |
| Tarmac | mean (SD) | 23.79 (19.04) | 15.86 (8.46) | **0.022** |
| Paving | mean (SD) | 1.85 (4.48) | 8.72 (6.2) | **<0.001** |
| All weather surface | mean (SD) | 1.13 (8.22) | 8.11 (7.89) | **<0.001** |
| Grass | mean (SD) | 69.07 (23.08) | 72.98 (12.73) | 0.915 |
| Bark | mean (SD) | 1.37 (2.52) | 0.23 (0.83) | 0.066 |
| Other | mean (SD) | 1.78 (4.87) | 5.15 (16.54) | 0.968 |
| What is the predominant landuse in the area around the school? | N(%) Residential | 47 (51.09%) | 26 (57.78%) | 0.480 |
|  | N(%) Open fields | 15 (16.3%) | 4 (8.89%) |  |
|  | N(%) Business | 0 (0%) | 0 (0%) |  |
|  | N(%) Mixture | 30 (32.61%) | 14 (31.11%) |  |
| ^a^ Abbreviations used: N = number, med = Median, IQR = inter quartile range (25^th^ centile-75^th^ centile), S disagree/S agree = Strongly disagree/Strongly agree | | | | |
